# Spatial origin of the extracellular ATP‐induced cytosolic calcium signature in *Arabidopsis thaliana* roots: wave formation and variation with phosphate nutrition

**DOI:** 10.1111/plb.13427

**Published:** 2022-05-08

**Authors:** E. Matthus, K. A. Wilkins, A. Mohammad‐Sidik, Y. Ning, J. M. Davies

**Affiliations:** ^1^ 2152 Department of Plant Sciences University of Cambridge Cambridge UK; ^2^ Leibniz Centre for Agricultural Landscape Research (ZALF) Müncheberg Germany

**Keywords:** *Arabidopsis*, calcium, extracellular ATP, phosphate, root, signature, wave

## Abstract

- Extracellular ATP (eATP) increases cytosolic free calcium ([Ca^2+^]_cyt_) as a specific second messenger ‘signature’ through the plasma membrane DORN1/P2K1 receptor. Previous studies revealed a biphasic signature in *Arabidopsis thaliana* roots that is altered by inorganic phosphate (Pi) deprivation. The relationship between the two phases of the signature and possible wave formation have been tested as a function of Pi nutrition.
- The bioluminescent aequorin and intensiometric GCaMP3 reporters were used to resolve the spatial origin of the eATP [Ca^2+^]_cyt_ signature in *Arabidopsis* root tips. Application of eATP only to the root apex allowed [Ca^2+^]_cyt_ wave resolution without the confounding effects of eATP delivery by superfusion.
- The first apical millimetre of the root generates the first [Ca^2+^]_cyt_ increase by eATP, regardless of nutritional status. The second increase occurs sub‐apically in the root hair zone, has some autonomy and is significantly reduced in Pi‐starved roots. A significant component of the Pi‐replete signature does not require DORN1/P2K1, but Pi‐starved roots appear to have an absolute requirement for that receptor. Application of eATP specifically to the root apex provides evidence for cell‐to‐cell propagation of a [Ca^2+^]_cyt_ wave that diminishes sub‐apically.
- The apex maintains a robust [Ca^2+^]_cyt_ increase (even under Pi starvation) that is the basis of a propagative wave, with implications for the ability of the root’s eATP signalling systems to signal systemically. Partial autonomy of the sub‐apical region may be relevant to the perception of eATP from microbes. eATP‐induced [Ca^2+^]_cyt_ increase may not have always have an obligate requirement for DORN1/P2K1.

## Graphical abstract

Extracellular ATP increases cytosolic free calcium at the root apex to induce a wave.

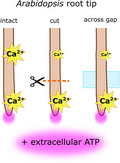

## INTRODUCTION

Extracellular ATP (eATP) is a plant cell regulator. It is involved in the maintenance of cell viability, growth and development (Chivasa *et al*. 2005; Clark *et al*. 2010; Wu *et al*. 2018), stomatal aperture regulation (Chen *et al*. 2018), abiotic stress responses (Hou *et al*. 2018), wounding and immunity (Choi *et al*. 2014a; Tripathi *et al*. 2018; Jewell *et al*. 2019; Nizam *et al*. 2019; Kumar *et al*. 2020). Application of ATP to plant tissue causes increases in free cytosolic Ca^2+^ ([Ca^2+^]_cyt_; Demidchik *et al*. 2003; Choi *et al*. 2014a; Matthus 2019a,b,c). Stimulus‐specific [Ca^2+^]_cyt_ elevations, or ‘signatures’, are decoded by specific suites of Ca^2+^‐binding proteins to direct transcriptional or physiological responses (Lenzoni *et al*. 2018). eATP can evoke [Ca^2+^]_cyt_ signatures in *Arabidopsis* seedlings (Jeter *et al*. 2004; Tanaka *et al*. 2010; Choi *et al*. 2014a; Chen *et al*. 2018; Lenzoni *et al*. 2018), leaves (Tanaka *et al*. 2010; Matthus *et al*. 2019c; Mohammad‐Sidik *et al*. 2021) and roots (*e.g*. Demidchik *et al*. 2003, 2009; Tanaka *et al*. 2010; Costa *et al*. 2013; Shi *et al*. 2015; Kelner *et al*. 2018; Matthus *et al*. 2019a‐c; Krogman *et al*. 2020; Waadt *et al*. 2020; Mohammad‐Sidik *et al*. 2021). Studies on *Arabidopsis* seedlings and roots suggest that eATP‐induced [Ca^2+^]_cyt_ elevation is totally reliant on the plasma membrane DORN1 (Does not Respond to Nucleotides1)/P2K1 eATP receptor (Choi *et al*. 2014a; Chen *et al*. 2018; Matthus *et al*. 2019c). This Ca^2+^ signalling directs a transcriptional response through the Ca^2+^‐dependent CAMTA3 transcriptional regulator (Jewell *et al*. 2019).

The eATP‐induced [Ca^2+^]_cyt_ signature in *Arabidopsis* roots is influenced by inorganic phosphate (Pi) nutrition (Matthus *et al*. 2019a). Pi‐replete excised root tips sustained a biphasic [Ca^2+^]_cyt_ increase in response to eATP. In contrast, Pi‐starved excised root tips only supported a monophasic eATP‐induced [Ca^2+^]_cyt_ increase – the second phase of [Ca^2+^]_cyt_ increase was absent (Matthus *et al*. 2019a). These findings, from using aequorin as a luminometric reporter of [Ca^2+^]_cyt_, were corroborated by using the ratiometric [Ca^2+^]_cyt_ reporter Yellow Cameleon 3.6 (YC3.6) to allow spatial resolution. Pi‐replete intact roots sustained a biphasic eATP‐induced [Ca^2+^]_cyt_ increase, with the first increment at the apex followed by a second, sub‐apical increase. The second, sub‐apical [Ca^2+^]_cyt_ increase was absent in Pi‐starved roots (Matthus *et al*. 2019a). The mechanism for suppression of that second sub‐apical [Ca^2+^]_cyt_ increase as a consequence of Pi nutrition remains unknown, but at the phenomenological level it relates to iron (Fe) availability (Matthus *et al*. 2019a,b). The second sub‐apical [Ca^2+^]_cyt_ increase was restored in Pi‐starved roots by removing Fe from the growth medium (Matthus *et al*. 2019a).

As the transcriptional response to a [Ca^2+^]_cyt_ signature depends on its temporal phases (Lenzoni *et al*. 2018), it is important to understand whether the first eATP‐induced [Ca^2+^]_cyt_ elevation activates the second and to what extent the second increase is autonomous. This is also relevant to the phenomenon of ‘Ca^2+^ waves’, in which a [Ca^2+^]_cyt_ signal may be propagated along a tissue (Choi *et al*. 2014b; Evans *et al*. 2016; Nguyen *et al*. 2018) to evoke a distal response. To date, a [Ca^2+^]_cyt_ wave has been detected in *Arabidopsis* roots challenged with salt stress (Choi *et al*. 2014b; Evans *et al*. 2016) and a [Ca^2+^]_cyt_ wave has been suggested to occur in response to eATP (Costa *et al*. 2013; Matthus *et al*. 2019c; Krogman *et al*. 2020). Studies on eATP have been limited by superfusive eATP application to the whole root. In this study, the spatial origin of the root’s apical eATP‐induced [Ca^2+^]_cyt_ elevation has been examined further (using aequorin and the GFP‐based intensiometric [Ca^2+^]_cyt_ reporter GCaMP3; Vincent *et al*. 2017). These [Ca^2+^]_cyt_ reporters have also been used to test the relationship between the apical and sup‐apical eATP‐induced [Ca^2+^]_cyt_ elevations, as a function of Pi nutrition. In wave studies, a simple technique has allowed eATP application only to the apex, overcoming the limitation of superfusion.

## MATERIAL AND METHODS

### Plant material and growth conditions

*Arabidopsis thaliana* Col‐0 constitutively expressing cytosolic (apo)aequorin or GCaMP3 were as previously described (Matthus *et al*. 2019a,c). The *dorn1‐1* mutant constitutively expressing cytosolic (apo)aequorin was as described by Choi *et al*. (2014a) and Matthus *et al*. (2019a,c). Growth conditions were as described by Matthus *et al*. (2019a). Growth medium was half strength Murashige and Skoog with vitamins (Duchefa, Haarlem, Netherlands) and 0.8% (w/v) agar (Bacto agar, BD Biosciences, Wokingham, UK), pH 5.6; ‘half MS’. This contained 0.625 mM phosphate (‘full Pi’). A custom‐made MS without Pi was used for ‘zero Pi’ conditions (Duchefa, DU1072) and KCl substituted for missing potassium from KH_2_PO_4_ exclusion (Matthus *et al*. 2019a).

### Root growth assay

*Arabidopsis* Col and *dorn1‐1* (both constitutively expressing cytosolic (apo)aequorin) were grown on full or zero Pi medium for 11 days. Plates were scanned using an Epson scanner with 300 dpi resolution. ImageJ software with the NeuronJ plugin was used to quantify primary root length (the 8‐ to 11‐day interval).

### Aequorin luminometry

**Fig. 1:**
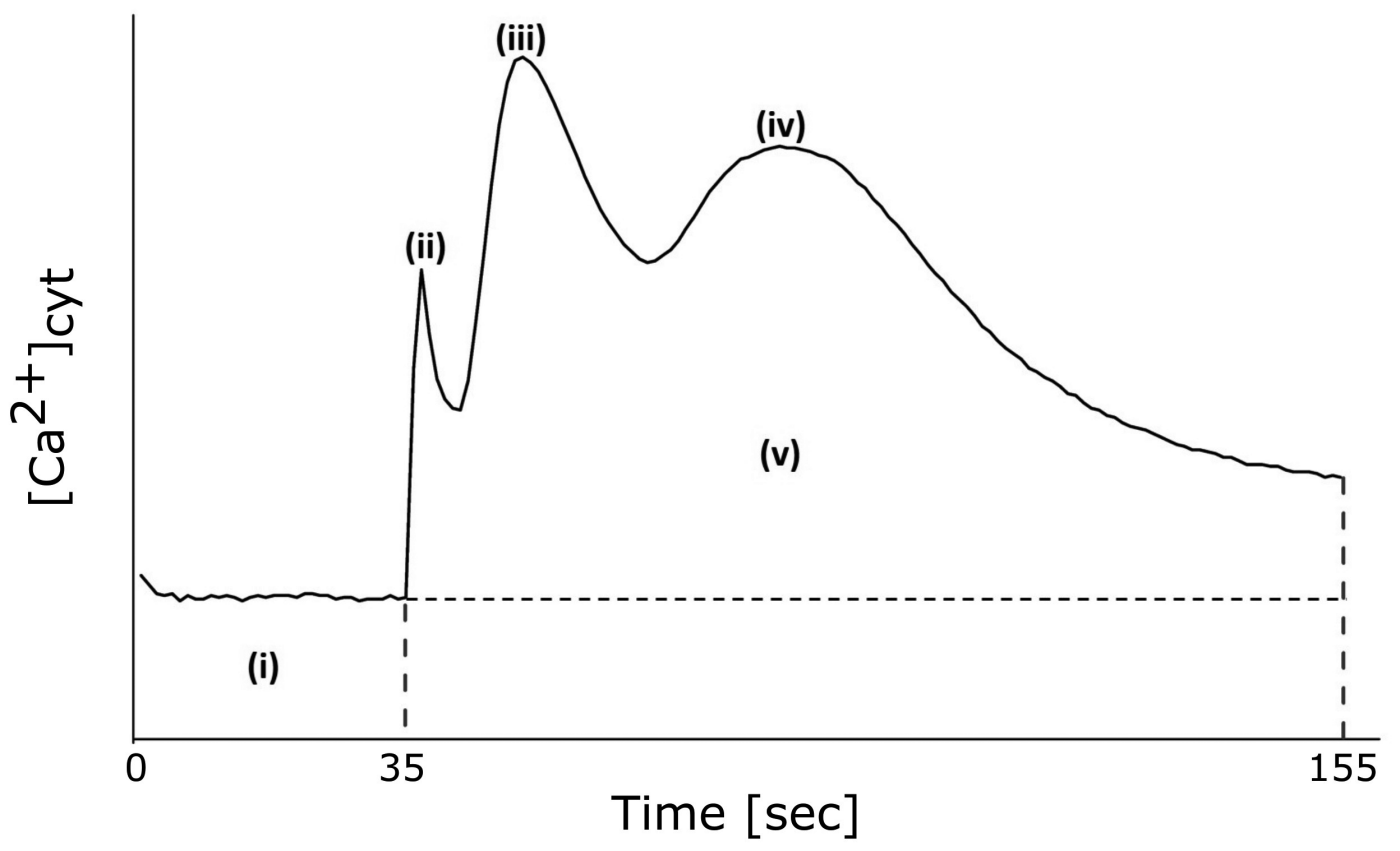
Schematic of [Ca^2+^]_cyt_ analysis from aequorin time‐course data. Each value was calculated with the average baseline value (i) subtracted. Touch peak was the highest value of the touch response because of mechanical stimulus from the treatment application (ii; 35–41 s or 35–155 s for control solution). Maximum Peak 1 (iii; 42–63 s) and Maximum Peak 2 (iv; 64–155 s) were the maximum values for each peak. Total [Ca^2+^]_cy_ accumulation (v) was obtained by integrating the area under the curve (AUC).

Excised 1‐cm long root tips (with or without the first apical millimetre excised) of 10‐day old seedlings were incubated overnight, in darkness at room temperature in 100 µl half MS containing 10 µM coelenterazine (NanoLight Technology, Pinetop AZ, USA), pH 5.6. with MES/Tris (Sigma, Darmstadt, Germany). Half MS medium had the same nutrient status *(i.e*. full Pi or zero Pi) as the plants were grown on. One root tip (1‐cm long with or without the first apical millimetre excised) was placed per well (containing 100 µl of the appropriate full Pi or zero Pi half MS) in a white 96‐well plate (Greiner Bio‐One, Kremsmuenster, Austria). Luminescence was recorded every second for 200 s (FLUOstar OPTIMA plate reader, BMG Labtech, Aylsebury, UK). After 35 s, 100 µl of control (full Pi or zero Pi half MS) or test solution (plus 1 mM ATP; Melford, Ipswich, UK) were added. Discharge solution (final concentration: 10% (v/v) ethanol, 1 M CaCl_2_) was injected after 120 s. [Ca^2+^]_cyt_ and changes in [Ca^2+^]_cyt_ were estimated according to Matthus *et al*. (2019a), in which peak maxima were detected in set timeframes. Total [Ca^2+^]_cyt_ mobilized was estimated as ‘Area Under the Curve’ (AUCLenzoni *et al*. 2018; Matthus *et al*. 2019a). A summary schematic is shown in Fig. 1.

### Imaging of GCaMP3

Roots or root tips from 10‐ to 11‐day‐old seedlings were placed on growth plates or, in ‘wave’ experiments, a root was placed across a gap in growth medium agar (Matthus *et al*. 2019c; Fig. S1). Recovery was for 5 to 10 min. Solution (3 µl) was applied by pipette; control solution (full Pi or zero Pi liquid half MS) ± 1 mM eATP. Imaging was with a Stereomicroscope M205 FA (Leica Microsystems, Wetzlar, Germany), with a DFC365FX camera (Leica) and a Sola SE365 light source (Lumencor, Beaverton OR, USA); excitation 470/40 nm, emission every 5 s at 525/50 nm, gain of 2.0 and 30× magnification. ImageJ Fiji was used to process GCaMP3 GFP signal intensities, fitting regions of interest (Roi) with the ‘ROI Manager’ tool. Z‐axis profiles were plotted for each Roi, and background signal was subtracted. Data normalization was as described by Vincent *et al*. (2017): ΔF/F_0_ = (F–F_0_)/F_0_, where F is the fluorescence signal and F_0_ is the baseline fluorescence signal. Maximal response was ΔF_max_/F_0_. Intensiometric false‐colour videos of response to control solution or ATP were compiled from a representative time series.

### Statistical analyses

Analyses used R software (www.r‐project.org; version 3.5.1). An anova, Welch two sample *t*‐test or paired Student’s *t*‐test was used to test for statistically significant differences, using a significance threshold of *P* < 0.05. When using an anova, the Tukey HSD *post‐hoc* test was employed to determine differences among the groups.

## RESULTS

### The first millimetre of the root apex is essential for the initial response to eATP

**Fig. 2:**
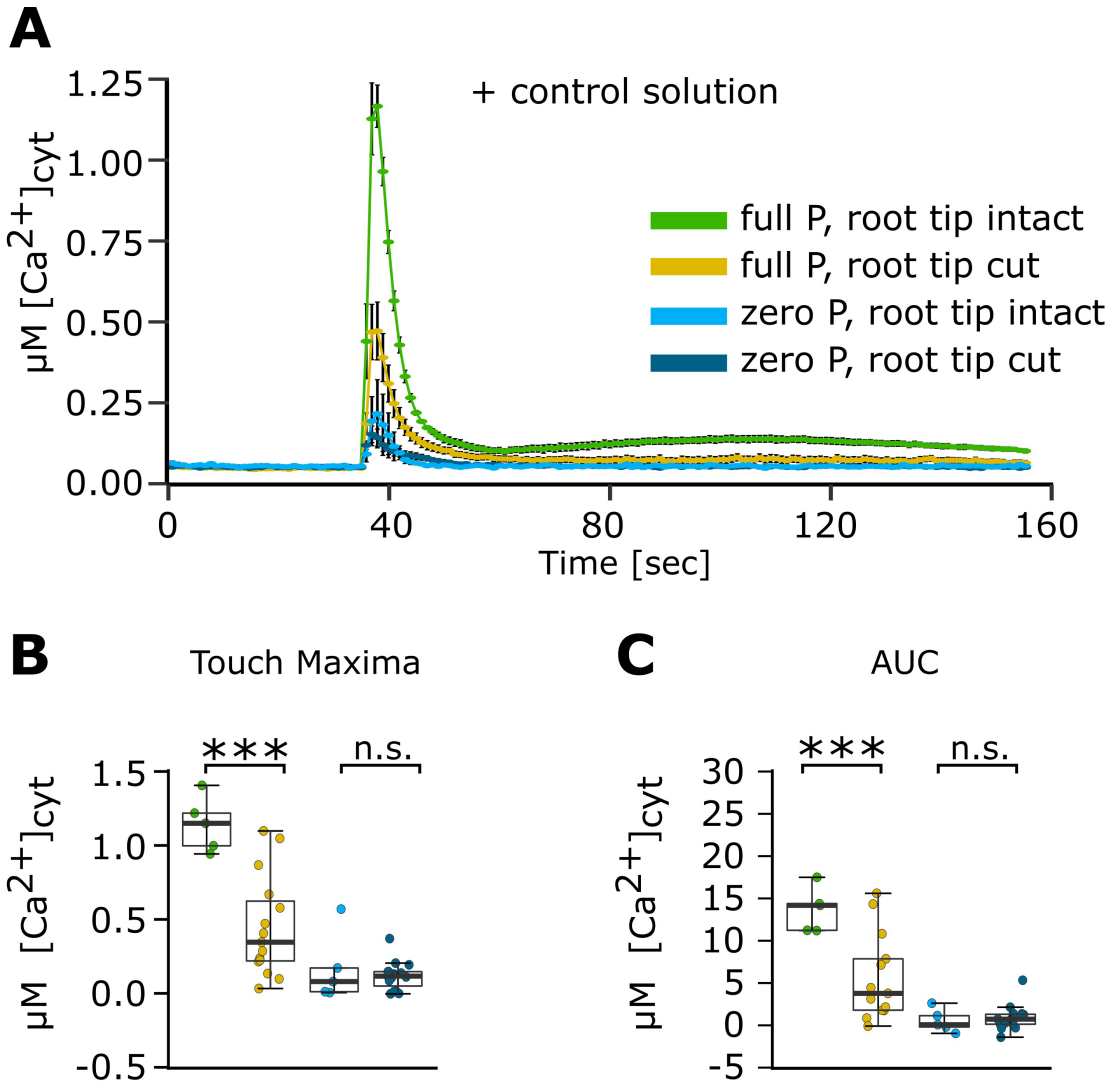
The [Ca^2+^]_cyt_ response of Pi‐replete and Pi‐starved intact or cut root tips to control solution. *Arabidopsis* Col‐0 aequorin‐expressing seedlings were grown on full or zero Pi medium. Root tips (1 cm; 'root tip intact’) or cut root tips (1 cm of root tip with the apical 1 mm cut off) were challenged with control solution applied at 35 s, and [Ca^2+^]_cyt_ was measured for 155 s. (A) Mechanical stimulation (caused by control solution): time course trace represents mean ± SEM from 2 independent trials, with n = 5 individual intact root tips, and 3 independent trials, with n = 15 individual cut root tips averaged per data point. Time course data were analysed for (B) touch maximum, (C) area under the curve (AUC), all baseline‐subtracted, with each dot representing an individual data point. In the boxplot, each dot represents an individual data point. The thick middle line denotes the median, separating the upper and lower half of the data; the hinges (box outline) denote median of the upper and the lower half of the data, respectively; bars denote entirety of data excluding outliers. anova with *post‐hoc* Tukey test was used to assess statistical differences. Significance ***P < 0.001, n.s. not significant.

**Fig. 3:**
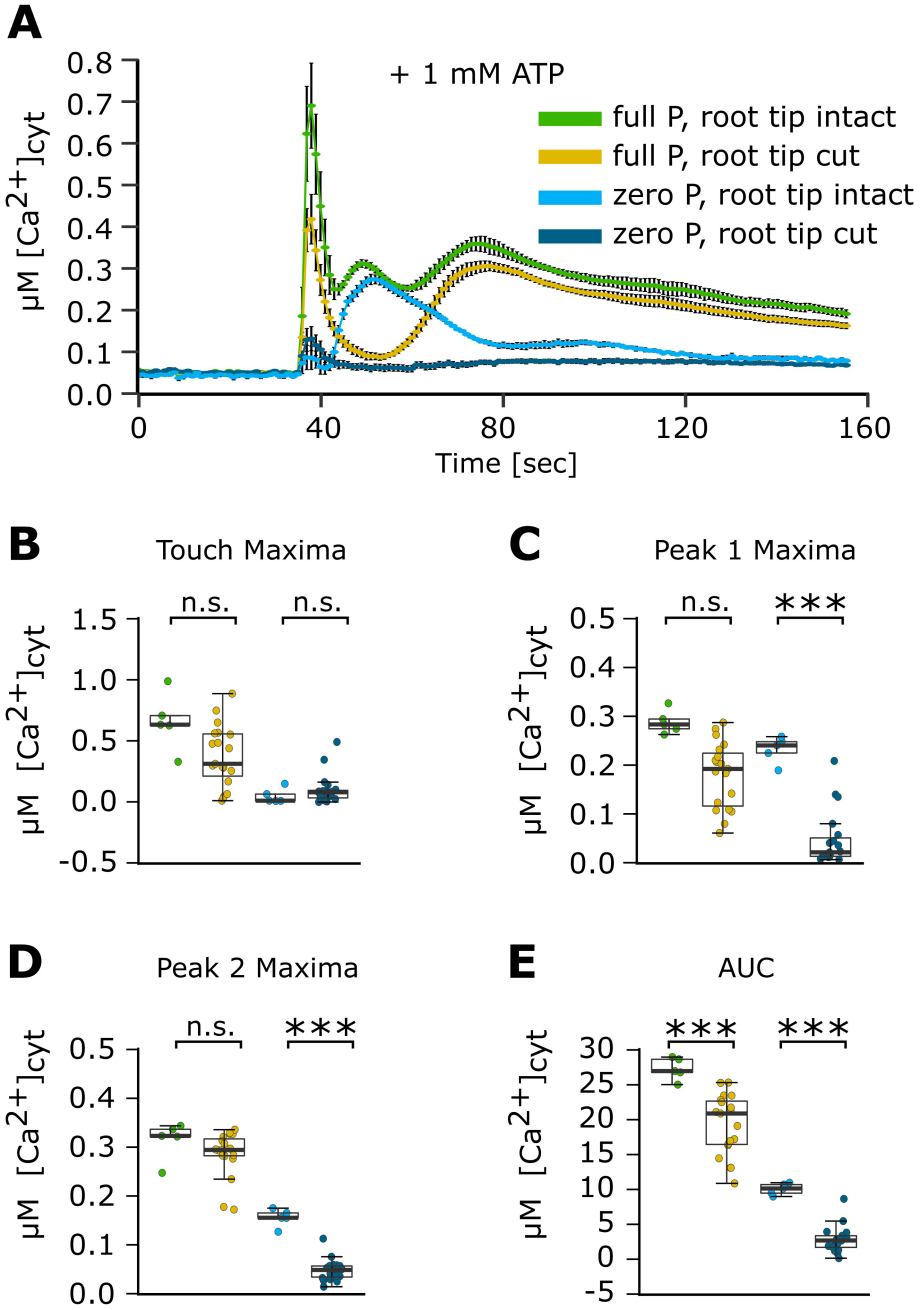
The [Ca^2+^]_cyt_ response of Pi‐replete and Pi‐starved intact or cut root tips to eATP. *Arabidopsis* Col‐0 aequorin‐expressing seedlings were grown on full or zero Pi medium. Root tips (1 cm; ‘root tip intact’) or cut root tips (1 cm of root tip with the apical 1 mm cut off) were challenged with 1 mM eATP applied at 35 s, and [Ca^2+^]_cyt_ was measured for 155 s. (A) eATP; time course trace represents mean ± SEM from 2 independent trials, with n = 5 individual intact root tips, and 3 independent trials, with n = 19 individual cut root tips averaged per data point. Time course data were analysed for (B) touch maximum, (C) Peak 1 maxima, (D) Peak 2 maxima and (E) area under the curve (AUC), all baseline‐subtracted, with each dot representing an individual data point. In the boxplot, each dot represents an individual data point. The thick middle line denotes the median, separating the upper and lower half of the data; the hinges (box outline) denote median of the upper and the lower half of the data, respectively; the bars denote entirety of data excluding outliers. anova with *post‐hoc* Tukey test was used to assess statistical differences. Significance: ***P < 0.001, n.s. not significant.

Root apical dissection was used to determine the spatial origin of the eATP‐induced [Ca^2+^]_cyt_ signature. Either the apical first centimetre of a root (termed ‘intact’) was used, or such root tips were further dissected by removal of approximately the first apical millimetre (termed ‘cut’). The first apical millimetre was removed as imaging with YC3.6 indicated that this region was responsible for the first eATP‐induced [Ca^2+^]_cyt_ elevation (Matthus *et al*. 2019a). In control experiments, solution added to intact root tips caused the characteristic monophasic ‘touch response’ (Matthus *et al*. 2019a, 2020; Mohammad‐Sidik *et al*. 2021). Maximum [Ca^2+^]_cyt_ touch response of full Pi‐grown ‘intact’ tips (Fig. 2A) was significantly larger than those (‘cut’) lacking the apical first millimetre (*P* < 0.001; Fig. 2B). Pi‐starved tips responded less to mechanical stimulation than full Pi‐grown root tips, regardless of whether the apical first millimetre was present (Fig. 2B). The same pattern was observed when analysing the area under the curve (AUC; estimating total [Ca^2+^]_cyt_ mobilized; Fig. 2C). eATP (1 mM) caused an initial [Ca^2+^]_cyt_ touch peak, followed by two eATP‐specific peaks in full Pi‐grown intact tips (Fig. 3A). Pi‐starved intact root tips showed the dampened [Ca^2+^]_cyt_ signature (Fig. 3A), with significantly diminished touch and peak 2 responses compared to full Pi‐grown intact root tips (touch, *P* < 0.001; peak 1, *P* = 0.9; peak 2, *P* < 0.001) and overall lower AUC (*P* < 0.001). These responses resembled the [Ca^2+^]_cyt_ signatures of intact aequorin‐expressing Col‐0 root tips to 1 mM eATP reported by Matthus *et al*. (2019a) using the same conditions.

Removing the apical 1 mm of the tip had no significant effect on touch response when eATP was added, regardless of growth regime (Fig. 3B). However, full Pi‐grown tips lacking the apical first millimetre also lacked the characteristic eATP‐induced peak 1. Mean [Ca^2+^]_cyt_ decreased to almost pre‐treatment baseline level before showing another increase in [Ca^2+^]_cyt_, which temporally aligned with the peak 2 observed in intact root tips from full Pi‐grown plants (Fig. 3A). Although the loss of peak 1 appeared striking (Fig. 3A), the effect was not statistically significant (*P* = 0.057; Fig. 3C). However, in the absence of the first peak, the method of detecting maxima in the sampling phase (Fig. 1) could report values from the end of the touch response or beginning of peak 2, thus overestimating the [Ca^2+^]_cyt_ of cut tips. When the mean ± SEM [Ca^2+^]_cyt_ of full Pi‐grown intact tips was compared to that of cut tips at 50 s (a time point within the mean peak 1 of full Pi‐grown intact tips), the response of cut tips was significantly lower than intact tips (cut, 0.04 ± 0.01 µM at 50 s; intact, 0.26 ± 0.01 µM at 50 s; *P* < 0.001; Fig. 3A), suggesting that the apical first millimetre of the root supports the first eATP‐induced [Ca^2+^]_cyt_ elevation in Pi‐replete conditions. Peak 2 maxima of full Pi‐grown cut tips were not significantly lower than intact tips, whether using phase analysis or comparing equivalent time points (Fig. 3D). AUC was significantly lower in full Pi‐grown cut root tips compared to intact root tips (Fig. 3E).

Removal of the apical first millimetre abolished the eATP‐specific [Ca^2+^]_cyt_ increase in zero Pi‐grown root tips (Fig. 3A). Mean [Ca^2+^]_cyt_ of cut tips was significantly lower than intact tips in the phases encompassing peaks 1 (Fig. 3C) and 2 (Fig. 3D). This was also the case when considering equivalent time points (*e.g*. peak 1 at 52 s, *P* < 0.001; peak 2 at 102 s, *P* < 0.005). AUC was significantly lower in zero Pi‐grown cut root tips compared to intact root tips (Fig. 3E). Overall, the data show that the apical first millimetre of the root tip is a key site for the generation of the first eATP‐induced [Ca^2+^]_cyt_ increase, regardless of Pi growth status. At this level of resolution, any dependency of peak 2 on peak 1 appears more likely in Pi‐starved roots.

### DORN1/P2K1 may not be the only eATP receptor in roots

**Fig. 4:**
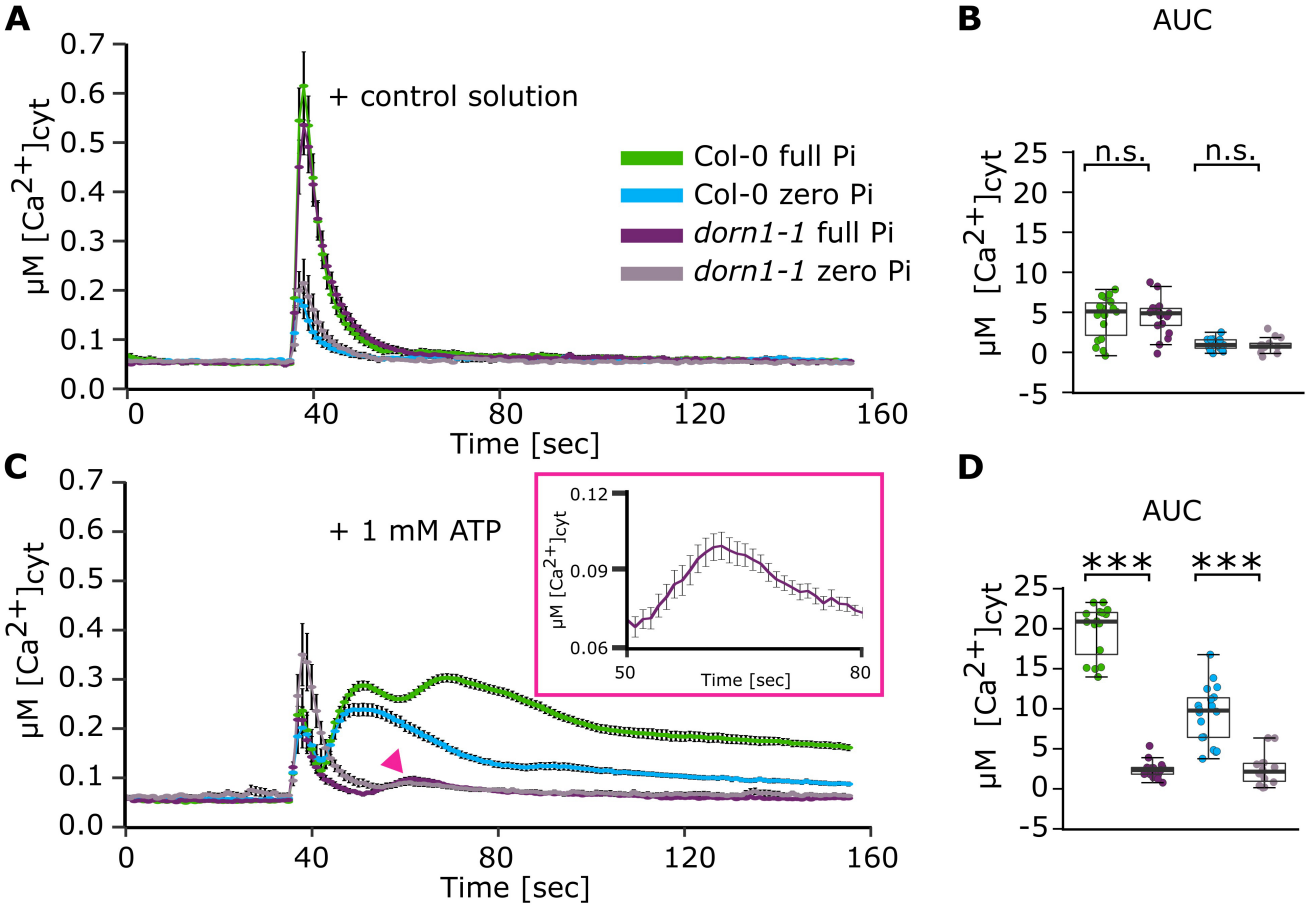
The [Ca^2+^]_cyt_ response to extracellular ATP requires DORN1/P2K1. Col‐0 and *dorn1‐1* were grown on full or zero Pi. Root tips (1 cm) were challenged at 35 s. (A) Control solution; mean ± SEM time course from 3 independent trials, with n = 13–18 individual root tips averaged per data point. Maximum [Ca^2+^]_cyt_ increase did not differ significantly between the genotypes for either Pi condition. (B) Time course data were analysed for area under the curve (AUC), baseline‐subtracted, with each dot representing an individual data point. Boxplot middle line denotes median. Comparisons shown are Col‐0 *versus dorn1‐1* for full Pi and zero Pi. (C, D) Responses to 1 mM eATP (3 independent trials, n = 16–18 individual root tips per growth condition and genotype). In (C) pink arrowhead points to the significant [Ca^2+^]_cyt_ increase in Pi‐replete *dorn1‐1*, an enlarged version of which is shown in the pink inset box. anova with *post‐hoc* Tukey test was used to assess statistical differences. Significance: ***P < 0.001, n.s. not significant.

The [Ca^2+^]_cyt_ response to eATP of Pi‐replete whole *Arabidopsis* roots appears to be wholly reliant on the DORN1/P2K1 receptor (Matthus *et al*. 2019c). Whether it is needed for the eATP response of Pi‐starved roots is unknown. In growth experiments, Pi deprivation impaired primary root growth of both Col and the *dorn1‐1* loss‐of‐function mutant, both constitutively expressing (apo)aequorin. Col mean ± SEM primary root length decreased significantly from 4.88 ± 0.06 cm in full Pi to 3.31 ± 0.06 cm in ‐Pi (*P* < 0.001; 106 and 100 roots, respectively, in 3 independent trials), while *dorn1‐1* decreased significantly from 3.81 ± 0.04 cm to 2.22 ± 0.05 cm, respectively (*P* < 0.001; 90 and 99 roots, respectively, in 3 independent trials). Although the *dorn1‐1* roots were significantly shorter than Col under full Pi (*P* < 0.001) and zero Pi (*P* < 0.01), the decrease in mean root length on Pi starvation of the two genotypes was similar (1.57 cm for Col, 1.59 for *dorn1‐1*), suggesting that growth inhibition was independent of DORN1/P2K1. The requirement of DORN1/P2K1 for the response of Pi‐starved and Pi‐replete root apices was tested here, using ‘intact’ 1 cm root tips of the *dorn1‐1* loss of function mutant (Fig. 4). In response to control solution, maximal [Ca^2+^]_cyt_ increase was significantly higher in full Pi‐grown root tips (Col‐0, 0.57 ± 0.07 µM; *dorn1‐1*: 0.49 ± 0.06 µM) than in zero Pi‐grown root tips (Col‐0, 0.13 ± 0.03 µM; *dorn1‐1*, 0.17 ± 0.05 µM) for both genotypes (Col‐0, *P* < 0.001; *dorn1‐1*, *P* = 0.001), but did not differ significantly between the genotypes for either Pi condition (Fig. 4A). AUC was significantly higher in full Pi‐grown root tips than zero Pi‐grown tips, regardless of genotype (Col‐0, *P* = 0.001; *dorn1‐1*, *P* = 0.002), and did not differ significantly between genotypes (Fig. 4B). In response to 1 mM eATP, Pi‐starved Col‐0 had a significantly diminished second eATP‐specific peak (full Pi, 0.26 ± 0.01 µM; zero Pi, 0.13 ± 0.01 µM, *P* < 0.001; Fig. 4C) and significantly lower AUC compared to Pi‐replete root tips (*P* < 0.001; Fig. 4D). The *dorn1‐1* response to eATP was dominated by the touch response, which did not differ significantly from Col‐0, regardless of Pi status (*P* ≥ 0.424; Fig. 4C). However, a significant increase in [Ca^2+^]_cyt_ was evident in Pi‐replete *dorn1‐1* after the touch response, within the 51–80 s analysis ‘window’ (0.11 ± 0.004 µM, *P* < 0.001, paired *t*‐test; Fig. 4C inset). Pi‐starved *dorn1‐1* lacked this response. These results point to a DORN1/P2K1‐independent pathway in Pi‐replete root tips that is lost under Pi deprivation.

### Phosphate‐starved roots are impaired in sub‐apical [Ca^2+^]_cyt_ increase in response to eATP measured with GCaMP3

**Fig. 5:**
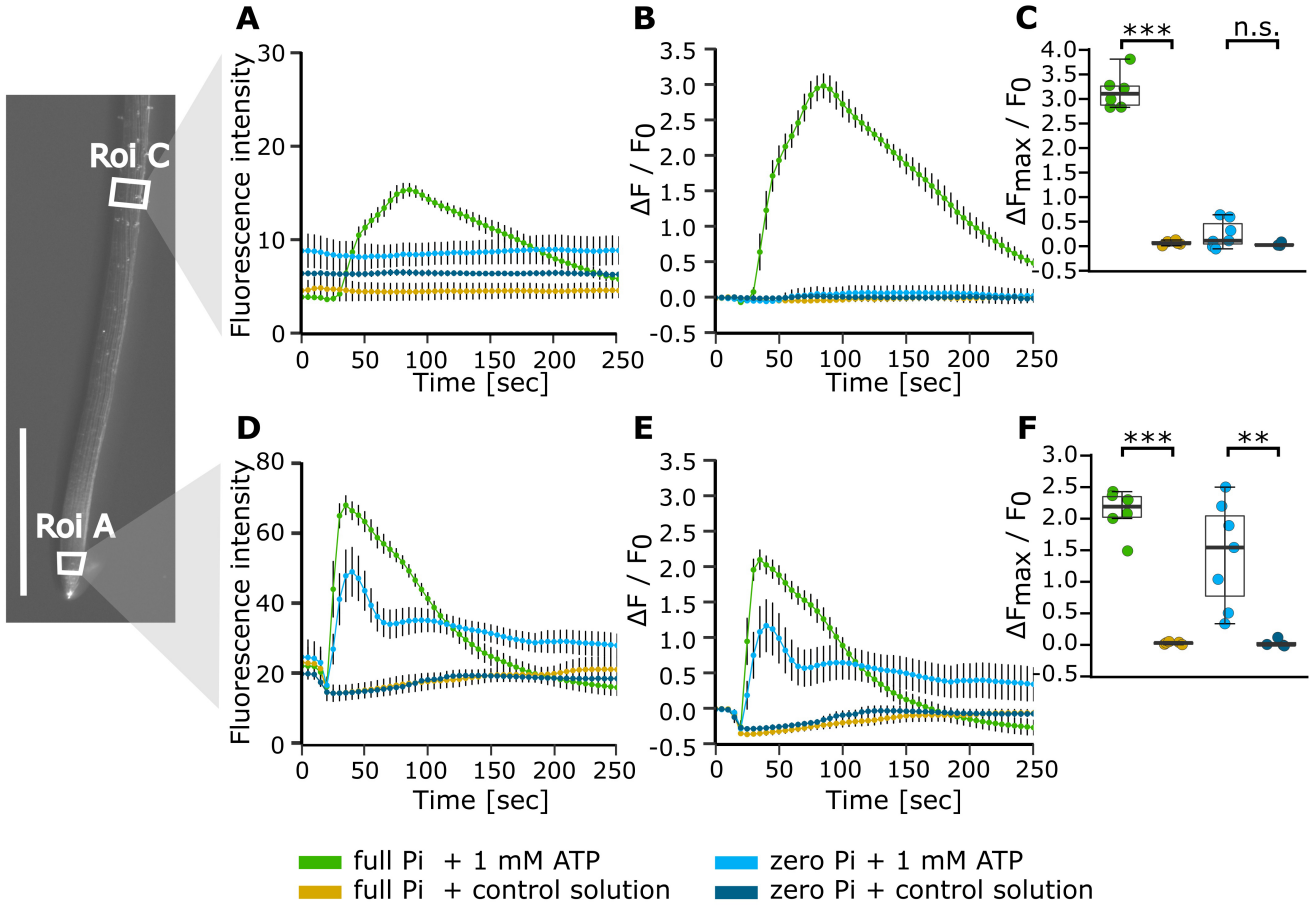
The [Ca^2+^]_cyt_ response to eATP in specific regions of root tips using an intensiometric reporter. *Arabidopsis* Col‐0 expressing cytosolic GCaMP3 was grown on either full or zero Pi medium for 10 days. Control or 1 mM eATP treatment solution was applied 20 s after the start of image acquisition to the root tip of a seedling resting on gel‐based growth medium, then imaged for 250 s in total. On the left, example root tip with annotated regions of interest (‘Roi’, white boxes), scale bar: 1 mm. (A, B): Roi C; (D, E): Roi A. (A, D) Mean GFP fluorescence intensity ± SEM, background subtracted, and (B, E) normalized GFP fluorescence (ΔF/F_0_) ± SEM; data from 3 independent trials, with n = 4–7 individual roots per growth condition and treatment. (C, F) Extracted normalized fluorescence maxima (ΔF_max_/F_0_). Boxplot thick line denotes median. anova with *post‐hoc* Tukey test was used to assess statistical differences. Significance: ***P < 0.001, n.s. not significant.

To afford spatial resolution of [Ca^2+^]_cyt_ changes, Col‐0 expressing cytosolic GCaMP3 was challenged with control solution (Videos S1 and S2) or 1 mM eATP (Videos S3 and S4). Two Roi (‘Region of interest’) were then analysed to assess spatial differences in the response to eATP (Fig. 5). The apical ‘Roi A’ was within the first millimetre of the apex and ‘Roi C’ was distal at 2.5 mm. Control solution led to little change in fluorescence (Fig. 5A–F). There were no significant differences in normalized fluorescence maxima between Pi‐grown and Pi‐starved roots in Roi A (*P* = 0.999) or Roi C (*P* = 0.998; Fig. 5C,F). Full Pi‐grown root tips showed a significant fluorescence increase in Roi A with 1 mM eATP (compared to control) that reached its maximum amplitude within 10 s (Fig. 5D–F; normalized fluorescence maxima, *P* < 0.001). After a time‐lag of approximately 50 s, a significant increase in fluorescence maximum was found in Roi C (Fig. 5A–C; *P* < 0.001), with the response to eATP being significantly stronger in Roi C compared to Roi A (normalized fluorescence maxima, *P* < 0.001).

Zero Pi‐grown root tips also responded significantly more strongly to eATP than to control treatment in Roi A (Fig. 5D–F; normalized fluorescence maxima, *P* = 0.002). Although the kinetics of the response were altered in zero Pi‐grown roots (Fig. 5D,E), the eATP‐induced normalized maximum in Roi A was not significantly different from full Pi‐grown roots (*P* = 0.118). In contrast to full Pi‐grown roots, those grown without Pi showed a weak response to eATP treatment in Roi C (Fig. 5A–C; *P* = 0.58). Thus, these data confirm the observations from aequorin trials: the first millimetre of the apex supports the first eATP‐induced [Ca^2+^]_cyt_ increase, regardless of Pi nutritional status. The spatial resolution afforded by GCaMP3 resolves a secondary and sub‐apical eATP‐induced [Ca^2+^]_cyt_ increase, distal to the first millimetre, that is weaker in Pi‐starved roots.

### The sub‐apical eATP‐induced [Ca^2+^]_cyt_ increase is not fully autonomous and relies in part on the apical increase

**Fig. 6:**
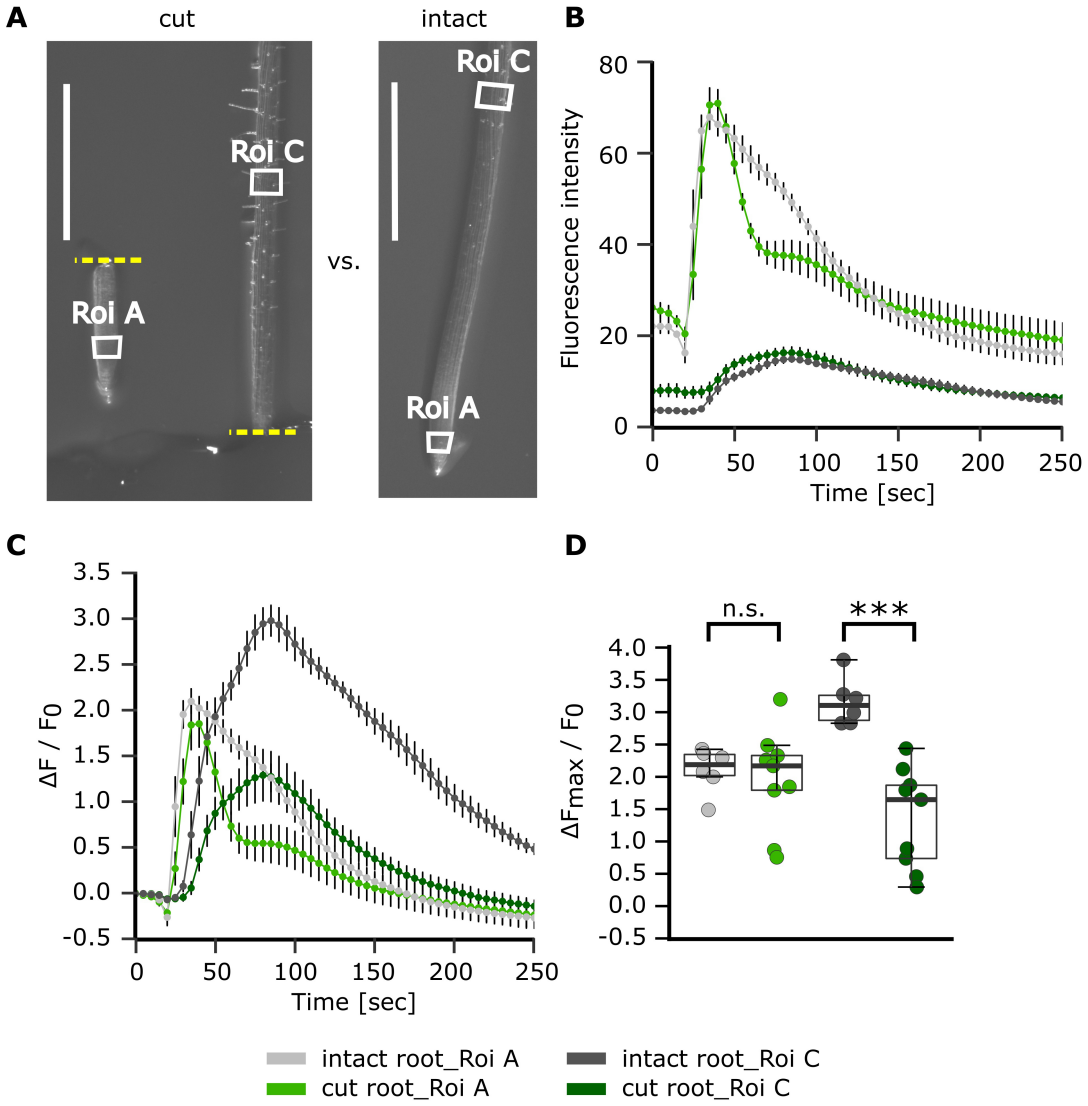
Root dissection reveals that the sub‐apical [Ca^2+^]_cyt_ response to eATP is not fully autonomous. *Arabidopsis* Col‐0 expressing the cytosolic GCaMP3 was grown on half MS growth medium (full Pi). Primary roots of 10‐day‐old seedlings were modified prior to the assays by excising 0.8–1.0 mm of apical root tip (‘cut root’) or left as ‘intact root’. At 20 s after the start of image acquisition, control or 1 mM eATP solution was applied to the root tip (and stump), which were then imaged for 250 s in total. (A) Root micrographs depicting cut root (yellow dashed line indicates site of cut, with root tip being placed next to root stump), and intact root, including regions of interest used for analysis (Roi A, Roi C; indicated by white boxes). Scale bar: 1 mm. (B) Mean GFP fluorescence intensity ± SEM, background subtracted, and (C) normalized GFP fluorescence (ΔF/F_0_) ± SEM; data from 3 independent trials, with n = 6–9 individual roots per root modification. (D) Extracted normalized fluorescence maxima (ΔF_max_/F_0_) of individual Roi, each dot represents an individual data point, boxplot thick line denotes median. anova with *post‐hoc* Tukey test was used to assess statistical differences. Significance: ***P < 0.001, n.s. not significant.

Full Pi‐grown GCaMP3‐expressing roots were dissected to investigate whether the first apical millimetre influenced the magnitude of the eATP‐induced sub‐apical second [Ca^2+^]_cyt_ peak. The apical first millimetre was excised and imaged alongside the remaining distal root ‘stump’, so that they received treatment simultaneously (Video S5), then compared with intact roots. Roi A was positioned at the apical root tip, Roi C was positioned 2.5 mm from the apical root tip (white boxes in Fig. 6A). After a slight decrease in fluorescence due to treatment application at 20 s, 1 mM eATP led to an immediate and strong increase in fluorescence intensity in Roi A of both intact and cut roots (Fig. 6B). While intact root Roi A supported a broad monophasic increase (grey trace; Fig. 6B), cut root tip Roi A exhibited a much narrower peak, followed by a smaller shoulder (light green trace; Fig. 6B,C). Nevertheless, normalized maximum response in Roi A was not significantly affected by apical excision (Fig. 6D; *P* = 0.975). eATP triggered a more gradual fluorescence increase in Roi C, with apical excision’s having little effect on time‐course (Fig. 6B,C). However, intact roots had a significantly higher normalized fluorescence maximum in Roi C (Fig. 6D; *P* < 0.001). These results suggest that sub‐apical regions can respond directly to eATP but, at the finer spatial resolution afforded by GCaMP3 compared to aequorin, part of their [Ca^2+^]_cyt_ increase is determined by the initial [Ca^2+^]_cyt_ increase at the apex.

### Transmission of an eATP‐induced [Ca^2+^]_cyt_ signal from the apex in Pi‐starved roots

**Fig. 7:**
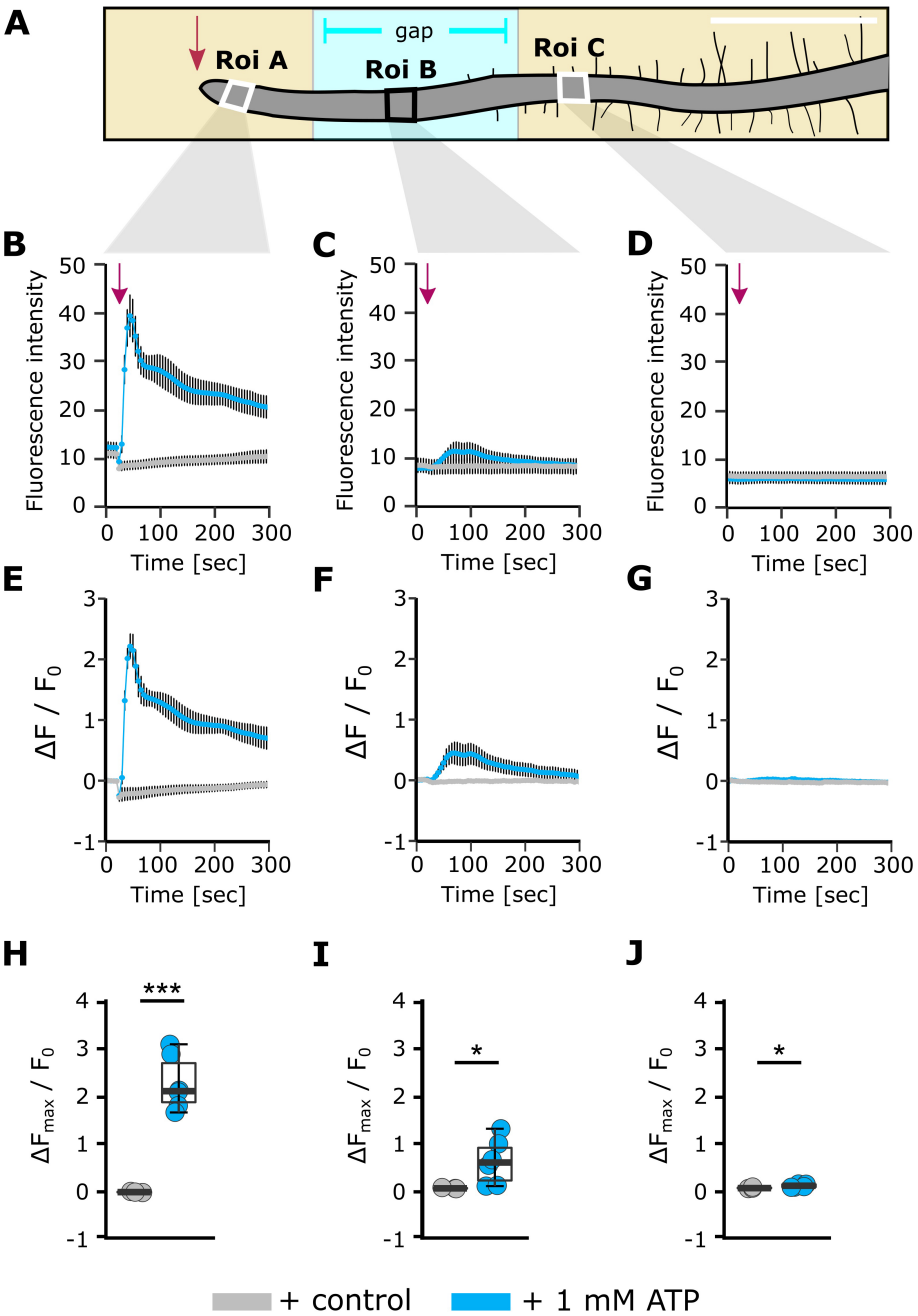
Localized eATP application to the root tip causes progressive [Ca^2+^]_cyt_ increases in the root. (A) Schematic of a 10‐day‐old *Arabidopsis* Pi‐starved Col‐0 seedling (expressing cytosolic GCaMP3) placed across a gap in the growth medium agar (Fig. S1). Annotated regions of interest (Roi) were used for analysis, scale bar: 1 mm. At 20 s after the start of image acquisition, 3 μl of control or 1 mM eATP solution was applied to the root tip (indicated by arrow), then imaged for a total 495 s. (B, C, D) Mean GFP fluorescence intensity±SEM, background subtracted, and (E, F, G) normalized GFP fluorescence (ΔF/F_0_) ± SEM for Roi A, B and C, respectively. Data are from 3 independent trials, with n = 3 individual roots for control treatments per growth condition, and n = 6–9 individual roots per eATP treatment and growth condition. (H, I, J) Extracted normalized fluorescence maxima (ΔF_max_/F_0_) for Roi A, B and C, respectively. Each dot represents an individual data point, middle line denotes median. Significance (*P‐*values, Welch two sample *t*‐test*)* in H–J: ***P < 0.001, *P < 0.05, n.s. >0.05.

The finding of a semi‐autonomous sub‐apical [Ca^2+^]_cyt_ increase in response to superfusion with eATP necessitated application of eATP to the apex only. This allowed further tests of the relationship between apical and sub‐apical [Ca^2+^]_cyt_ elevations in a possible ‘wave’. A GCaMP3‐expressing Pi‐starved root was placed over an air gap in the underlying agar medium, thus isolating the regions on either side of that gap from addition of eATP (Fig. S1). The air gap began approximately 1 mm from the root apex, such that the apical tissue likely to generate the first [Ca^2+^]_cyt_ peak was in contact with the agar. Three Roi were set: at the apex (Roi A), over the air gap (Roi B) and in the mature zone (Roi C at 2.5 mm from the apex; Fig. 7A). Control solution had no effect (Fig. 7B‐J). eATP applied to the apex caused a rapid and significant increase in fluorescence with no recovery to baseline level (Roi A, *P* < 0.001; Fig. 7B, E, H). Smaller but significant transient fluorescence increases (normalized maxima compared to control) were detected later in the root section over the air gap (Roi B; Fig. 7C–J; *P* = 0.04) and in the mature zone (Roi C; Fig. 7C–J; *P* = 0.02). Roi C was in the equivalent position to Roi C in the superfusion experiment (2.5 mm from apex; Fig. 5A–C). In the latter, eATP did not induce a significant [Ca^2+^]_cyt_ elevation (due to variation) in Roi C, but it is important to note that the mean peak normalized increase in this region was five times larger under superfusion than when eATP was applied only to the apex (mean ± SEM superfusion Roi C, 0.25 ± 0.11; apical application and resultant response in Roi C, 0.05 ± 0.01). As the mature zone was not exposed to eATP, the results suggest a cell‐to‐cell propagative mechanism for [Ca^2+^]_cyt_ over short distances.

## DISCUSSION

Root apices are key to the plant’s sensing of and adaptive responses to heterogeneity in soil conditions. Pi starvation results in altered abiotic stress‐induced [Ca^2+^]_cyt_ signatures in *Arabidopsis* root tips (Matthus *et al*. 2019a). These include the response to mechanical stress: Pi‐starved root tips or whole roots expressing cytosolic aequorin were found to have a lower [Ca^2+^]_cyt_ touch response than Pi‐replete root tips (Matthus *et al*. 2019a, 2020). This phenomenon was also observed here (Fig. 2). Mechanical stimulation can evoke a spatially complex [Ca^2+^]_cyt_ signature, and sensitivity varies along the root (Monshausen *et al*. 2009; Krogman *et al*. 2020). The simple experiment of removing the first apical millimetre of full Pi‐grown root tips significantly reduced (but did not abolish) their touch response, helping to site the origin of this mechanically‐induced [Ca^2+^]_cyt_ signature. Pi‐starved root tips were unaffected by excision of the first apical millimetre, suggesting that their [Ca^2+^]_cyt_ touch response originates more sub‐apically. These Pi‐dependent changes in the touch response were not evident in the trials using GCaMP3 as the [Ca^2+^]_cyt_ reporter. This may be due to the differences in mechanical stress experienced by the root tip as a consequence of different solution application methods (pump injection for aequorin *versus* pipette application for GCaMP3), or differences in experimental handling/conditions that could change the root’s touch sensitivity. It is unlikely to reflect the sensitivity of GCaMP3 as it has a far higher Kd for Ca^2+^ (405 to 660 nM) than aequorin (7 to 13 µM). The mechanistic basis of the dampened touch response of Pi‐starved aequorin‐expressing roots could have a variety of origins, including downregulation of mechano‐sensitive Ca^2+^ influx pathways involving the plasma membrane proteins MCA1 (Mid1‐Complementing Activity1; Okamoto *et al*. 2021), AtPiezo (Fang *et al*. 2021) or DEK1 (Defective Kernel 1; Tran *et al*. 2017). It would be interesting to determine whether Pi‐starved roots also have aberrant touch‐induced hormonal responses, such as impaired regulation of ethylene and jasmonic acid, and whether these relate to touch‐induced changes in root system architecture (Chehab *et al*. 2012; Jacobsen *et al*. 2021; Okamoto *et al*. 2021). This could be relevant to the challenge of breeding crops for compacted and Pi‐poor soils (Kolb *et al*. 2017).

The primary root apex is also a hotspot of extracellular ATP accumulation (Weerasinghe *et al*. 2009), and the abundance of the DORN1/P2K1 eATP receptor is higher there than in distal regions (Matthus *et al*. 2019c). The mechanical stress experienced as roots grow through soil is sufficient to promote further eATP accumulation, regulated by plasma membrane heterotrimeric G proteins (Weerasinghe *et al*. 2009). Salt, osmotic and cold stress can also increase eATP accumulation by roots (Dark *et al*. 2011; Lang *et al*. 2014; Deng *et al*. 2015), as can wounding and endophyte colonization (Dark *et al*. 2011; Nizam *et al*. 2019). eATP appears able to stimulate root adaptive responses, affording protection against abiotic stress (Lang *et al*. 2014), limiting colonization of the endophyte *Serendipita indica* (Nizam *et al*. 2019) and enhancing protection against pathogens (Kumar *et al*. 2020). Using excised root tips to study the eATP‐evoked [Ca^2+^]_cyt_ signature helps to negate any possible effects of changes in root architecture caused by Pi deprivation (Matthus *et al*. 2019a). Moreover, using tips rather than whole roots has permitted resolution of a small, but significant, DORN1/P2K1‐independent pathway to [Ca^2+^]_cyt_ elevation in Pi‐replete roots (Fig. 4C). This was lost under Pi‐starvation. DORN1‐P2K1 expression does not respond to Pi starvation (Lin *et al*. 2011; Lan *et al*. 2012; genevestigator.com), neither to nitrogen nor K^+^ deficiency (Kellermeier *et al*. 2014). However, the abundance of DORN1/P2K1 in roots has been reported to *increase* on Pi starvation (Lan *et al*. 2012). These data suggest that DORN1‐P2K1 remains operational under nutrient shortages to effect a robust eATP signalling system and that the impaired second eATP‐induced [Ca^2+^]_cyt_ response of Pi‐starved roots may not simply be due to lack of the receptor in the sub‐apical region. That the putative DORN1‐P2K1‐independent pathway was Pi‐sensitive may help resolve its mechanistic basis. Recently, a DORN1/P2K1‐independent [Ca^2+^]_cyt_ elevation was observed in leaves (Matthus *et al*. 2019c), and results presented here in roots augment the findings of Zhu *et al*. (2018, 2020) that eATP effects on roots may not always require DORN1/P2K1.

It is clear from use of both aequorin and GCaMP3 trials that Pi‐starvation impairs the eATP‐induced [Ca^2+^]_cyt_ response, in agreement with a previous study using aequorin and YC3.6 (Matthus *et al*. 2019a). Specifically, the second eATP‐induced [Ca^2+^]_cyt_ elevation is consistently weakened. Excision experiments using aequorin revealed that the first apical millimetre of Pi‐replete root tips is essential for the first eATP‐induced [Ca^2+^]_cyt_ peak and seemingly the entire [Ca^2+^]_cyt_ response of Pi‐starved root tips (Fig. 1). That first apical millimetre includes the root cap, meristem and elongation zone; as previously stated, this is a region of high eATP accumulation and DORN1/P2K1 eATP receptor abundance. The spatial resolution afforded by GCaMP3 showed that the kinetics of the [Ca^2+^]_cyt_ response to eATP within the first apical millimetre was influenced by Pi nutrition (Fig. 5). This, in addition to overall change in [Ca^2+^]_cyt_ signature, could be relevant to any downstream transcriptional response, as modelling suggests that decay time strongly influences those events (Lenzoni *et al*. 2018). It would be interesting to determine whether such an altered transcriptional response governed by the first eATP‐induced [Ca^2+^]_cyt_ peak in that first apical millimetre could be involved in the altered root growth that occurs on Pi starvation, although preliminary experiments here suggested that inhibition of primary root growth was independent of DORN1/P2K1. Excision experiments with GCaMP3 showed that sub‐apical regions can respond directly to eATP, indicating a level of autonomy, but with a weaker response than in intact roots. This autonomy could be important for the root’s ability to sense eATP emanating from microbes in the vicinity. The use of an air gap in the underlying growth medium permitted application of eATP only to the apex, allowing the effect of Pi starvation on the sub‐apical response to be resolved clearly and without any confounding effects that excision or superfusion with eATP might cause. Although small, a significant [Ca^2+^]_cyt_ increase occurred in the mature zone (beyond the air gap) when eATP was added to the apex. This is consistent with a [Ca^2+^]_cyt_ ‘wave’ that propagates from the apex but gradually diminishes. As the GCaMP3 reporter is comparably insensitive to subtle changes in [Ca^2+^]_cyt_, the data presented likely underestimate the extent of any systemic [Ca^2+^]_cyt_ signal propagation compared to the more sensitive reporter (YCnano‐65) used in studying salt‐induced‐waves (Choi *et al*. 2014b). Nevertheless, the intensiometric GCaMP3 reporter promises ease of imaging using a less specific microscope set‐up.

The salt‐induced [Ca^2+^]_cyt_ wave is underpinned by RBOHD and the vacuolar Ca^2+^ release channel TPC1 (Two Pore Channel1; Evans *et al*. 2016). As DORN1/P2K1 interacts with RBOHD in guard cells (Chen *et al*. 2018), and is involved in eATP regulation of root hair growth (Clark *et al*. 2010), this NADPH oxidase may reprise its role in the root eATP‐induced wave. As with the salt wave, the plasma membrane Ca^2+^ channels that contribute to initiation and propagation need to be identified at the genetic level. It has been suggested that DORN1/P2K1 could generate cyclic nucleotides that could activate Cyclic Nucleotide‐Gated Channels (CNGC) (Sun *et al*. 2021). CNGC14 has been tested in Pi‐replete conditions and found not to be involved (Shih *et al*. 2015). However, CNGC2, CNGC4 and CNGC6 have since been shown to contribute to the root eATP‐induced [Ca^2+^]_cyt_ elevation in Pi‐replete conditions (Duong *et al*. 2022; Wang *et al*. 2022). Among the Glutamate Receptor‐Like family (GLR), GLR3.3 and GLR3.6 are involved in leaf wounding and signal propagation (Vincent *et al*. 2017). It is tempting to place them in the eATP‐induced signature and wave, particularly if (as in tea) Pi deficiency decreases glutamate (Ding *et al*. 2017). This might help explain the diminution of the sub‐apical component. As yet, no putative Ca^2+^ channels have been found to be downregulated at the protein level in response to Pi deprivation (*e.g*. Lan *et al*. 2012). Neither have they been found in phosphorylation studies of Pi deprivation (Duan *et al*. 2013). Annexins may have Ca^2+^ transport capacity but are multifunctional proteins (Laohavisit & Davies, 2010). *Arabidopsis* Annexin4 can support an eATP‐induced [Ca^2+^]_cyt_ elevation when expressed in HEK cells (Ma *et al*. 2019), while Annexin1 supports a significant proportion of the whole root [Ca^2+^]_cyt_ response to eATP (Mohammad‐Sidik *et al*. 2021). Identification of the components involved will greatly improve understanding of the eATP‐induced signature and wave as a function of Pi nutrition, helping to elucidate the downstream consequences in roots and potentially shoots.

## Supporting information


**Fig S1.** Brightfield image of a phosphate‐starved *Arabidopsis* root laid across an air gap on an agar plate to carry out GCaMP3 ‘wave’ experiments.

**Video S1.** Full Pi‐grown Col0 root expressing GCaMP3 responding to control solution added at 20 s after start of image acquisition; scale bar, 1 mm.

**Video S2.** Zero Pi‐grown Col0 root expressing GCaMP3 responding to control solution added at 20 s after start of image acquisition; scale bar, 1 mm.

**Video S3.** Full Pi‐grown Col0 root expressing GCaMP3 responding to 1 mM ATP added at 20 s after start of image acquisition; scale bar, 1 mm.

**Video S4.** Zero Pi‐grown Col0 root expressing GCaMP3 responding to 1 mM ATP added at 20 s after start of image acquisition; scale bar, 1 mm.

**Video S5.** Full Pi‐grown Col0 root expressing GCaMP3, with apex excised and placed next to remaining ‘stump’, responding to 1 mM ATP added at 20 s after start of image acquisition; scale bar, 1 mm.
