# Supplementary material for: Spatial origin of the extracellular ATP‐induced cytosolic calcium signature in Arabidopsis thaliana roots: wave formation and variation with phosphate nutrition
Source: Plant Biol (Stuttg). 2022 May 8;24(5):863–73. doi: 10.1111/plb.13427 (PMC10286661; doi:10.1111/plb.13427)
Supplement: Supplementary file 1 — Fig S1. Brightfield image of a phosphate‐starved Arabidopsis root laid across an air gap on an agar plate to carry out GCaMP3 ‘wave’ experiments. [file PLB-24-863-s006.docx]

**Supplementary Figure**


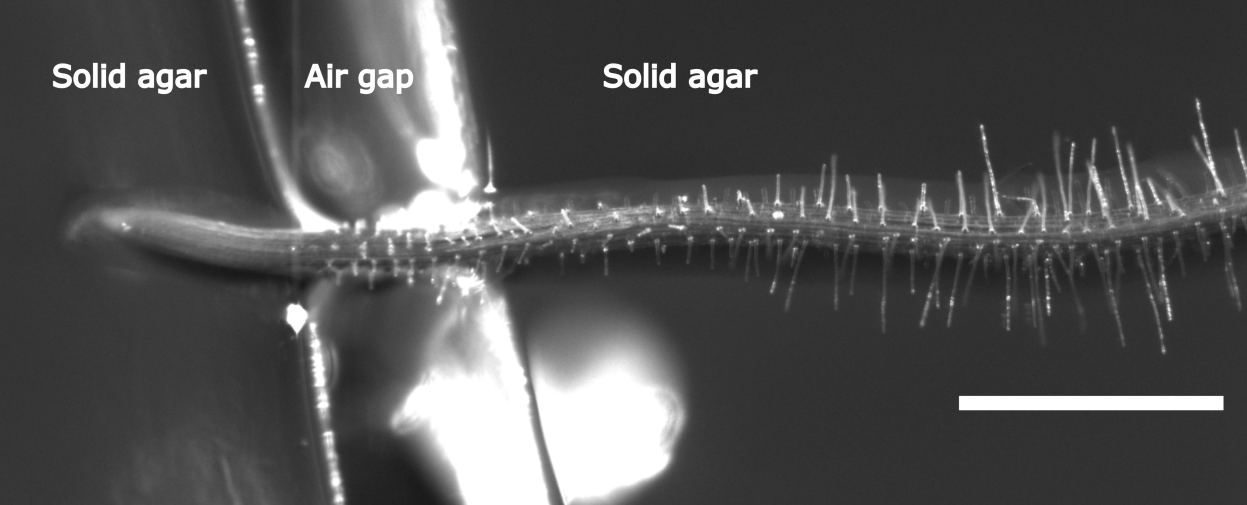


**Fig. S1.** Brightfield image of a phosphate-starved *Arabidopsis* root laid across an air-gap on an agar plate to carry out GCaMP3 “wave” experiments. Scale bar: 1mm.
